# Pharmacological Dose-Effect Profiles of Various Concentrations of Humanised Primary Bile Acid in Encapsulated Cells

**DOI:** 10.3390/nano12040647

**Published:** 2022-02-15

**Authors:** Armin Mooranian, Melissa Jones, Daniel Walker, Corina Mihaela Ionescu, Susbin Raj Wagle, Bozica Kovacevic, Jacqueline Chester, Thomas Foster, Edan Johnston, Jafri Kuthubutheen, Daniel Brown, Marcus D. Atlas, Momir Mikov, Hani Al-Salami

**Affiliations:** 1 The Biotechnology and Drug Development Research Laboratory, Curtin Medical School & Curtin Health Innovation Research Institute, Curtin University, Bentley, Perth, WA 6102, Australia; a.mooranian@curtin.edu.au (A.M.); melissa.a.jones@postgrad.curtin.edu.au (M.J.); daniel.walker1@postgrad.curtin.edu.au (D.W.); c.ionescu@postgrad.curtin.edu.au (C.M.I.); susbinraj.wagle@postgrad.curtin.edu.au (S.R.W.); bozica.kovacevic@postgrad.curtin.edu.au (B.K.); j.chester@graduate.curtin.edu.au (J.C.); thomas.p.foster@student.curtin.edu.au (T.F.); edan.johnston@graduate.curtin.edu.au (E.J.); 2 Hearing Therapeutics, Ear Science Institute Australia, Queen Elizabeth II Medical Centre, Nedlands, Perth, WA 6009, Australia; marcus.atlas@earscience.org.au; 3 Fiona Stanley Hospital, Murdoch, Perth, WA 6150, Australia; jafri.kuthubutheen@health.wa.gov.au; 4 Curtin Medical School, Curtin Health Innovation Research Institute, Curtin University, Bentley, Perth, WA 6102, Australia; daniel.brown2@curtin.edu.au; 5 Department of Pharmacology, Toxicology and Clinical Pharmacology, Faculty of Medicine, University of Novi Sad, Hajduk Veljkova 3, 21101 Novi Sad, Serbia; momir.mikov@mf.uns.ac.rs

**Keywords:** microencapsulation, diabetes mellitus, bile acids, chenodeoxycholic acid, pancreatic beta-cell line, inflammation

## Abstract

Bile acids (BA)s are known surfactants and well-documented to play a major role in food digestion and absorption. Recently, potential endocrinological and formulation-stabilisation effects of BAs have been explored and their pharmacological effects on supporting cell survival and functions have gained wide interest. Hence, this study aimed to explore the hyper-glycaemic dependent dose-effect of the BA chenodeoxycholic acid (CDCA) when encapsulated with pancreatic β-cells, allowing assessment of CDCA’s impacts when encapsulated. Four different concentrations of the BA were prepared, and viable cells were encapsulated and incubated for 2 days. Multiple analyses were carried out including confocal imaging, glucose-induced cellular mitochondrial viability indices, insulin production, inflammatory biomarker analyses and cellular bioenergetics measurements. There was a significant dose-effect with different concentrations of the BA, affecting cellular viability and antioxidant activities, cell functions and insulin release, inflammatory biomarkers, and cellular-bioenergetics at different oxidative stress levels. The results demonstrate that, when encapsulated, the BA CDCA exerts positive pharmacological effects at the cellular level, and such effects are concentration dependent.

## 1. Introduction

Bile acids (BA)s are amphipathic molecules, secreted by the liver, that play many biological roles, including absorption of dietary lipids. BAs have many promising properties which can be utilised in enhancing oral absorption. Such properties include their biocompatibility and ability to increase microcapsule survival within the gastrointestinal tract’s harsh environment [1,2].

Chenodeoxycholic acid (CDCA) is a primary BA which is the most potent agonist for the BA sensor farnesoid X receptor (FXR) [2,3,4]. CDCA is also a natural ligand of G protein-coupled receptor (TGR5). In diabetes patients, CDCA has been demonstrated to promote the release of glucagon-like peptide 1 (GLP-1), potentially through activation of TGR5. Mouse studies by Chen et al. showed CDCA improved glucose tolerance and regulated insulin levels whilst on a high fat diet. This result helped support the notion of CDCA’s use in metabolic disorder treatment, due to its regulatory effects [3,5].

The encapsulation of cells is a rapidly emerging process which offers significant potential in several areas. Implantation of such encapsulated cells may be utilised in the creation and development of functional bioartificial organs [3,6]. One key benefit of cell encapsulation and bioartificial organs is the protection of the cells from the host’s environment via a semipermeable encapsulation layer which is isolated from the immune system of the host. The semipermeable nature allows nutrient and waste exchange whilst simultaneously providing protection [7].

Whilst there are several examples of cell encapsulation, one area which is being heavily researched is the encapsulation of pancreatic β-cells. β-Cell encapsulation has potential use in the development of a bioartificial pancreas [8,9]. One cell line which may be useful is MIN6 pancreatic β-cells. MIN6 cells are derived from transgenic mice, and their cellular parameters make them a reliable and analogous model of human islets. Of particular benefit is MIN6s’ ability to be utilised as a comparable model in glucose-stimulated insulin release studies, including under hyperglycaemic conditions [10,11].

Creation of a functional pancreatic organ would offer great benefit in the treatment of diabetes mellitus, an ailment which affects a significant proportion of the global population and is a significant burden for health care systems [7,12]. In order to create such an organ, microcapsules with the most optimised characteristics would need to be created. Many potential excipients have been explored with this goal in mind.

Diabetes mellitus has been investigated for its links to both inflammation and oxidative stress. In terms of type 1 diabetes and inflammation, immunological involvement and inflammation have led to the potential for anti-inflammatory treatments to be of benefit [13]. Furthermore, a multitude of complications and side effects are proposed to be as a result of oxidative stress, in addition to proposed links between reactive oxygen species and pathogenesis of diabetes [14,15]. Hence, the use of CDCA may be beneficial as discussed below.

BAs such as CDCA have the potential to be useful drug excipients. CDCA has many properties which may assist in the microencapsulation of β-cells. CDCA has been shown to act as a permeation enhancer for hydrogels when used in oral delivery systems [16,17]. Furthermore, the endocrinological properties of CDCA may also be beneficial [18]. CDCA has also been shown to aid in cell survival by increasing resistance to apoptosis, which would be of assistance to microencapsulated cells [19].

Yan et al. have also shown in studies of CDCA that it has the potential for use as a treatment of osteoarthritis. In particular, this research highlighted that CDCA has significant anti-inflammatory properties [20]. In addition, Shaik et al. demonstrated that CDCA also has anti-inflammatory properties in tissues where FXR receptors are present. The authors show that this may be due to the anti-inflammatory properties of FXR, which are activated by CDCA treatment [21].

Several studies have been conducted utilising CDCA, showing its potential use in microencapsulation. These include Mathavan et al. who microencapsulated CDCA with sodium alginate (SA) for the delivery of the drug gliclazide. Their results indicated that CDCA improved the stability and physical characteristics of the produced microcapsules, as well as enhancing the release profiles of the drug gliclazide [16]. Mooranian et al. conducted similar studies with CDCA and SA, but with the drug probucol. The authors’ results demonstrated that CDCA improved the release profile of probucol, as well as the strength and stability of resultant microcapsules [22].

Other excipients useful for encapsulation include the polymer SA, which has strong safety and biocompatibility profiles and is an efficient solubilising agent. Historically, it has been popular for use in cell encapsulation, due to its stabilisation properties and ability to support cellular metabolism and functions [23,24,25,26].

Poly-L-ornithine (PLO) is a useful biomaterial in cell encapsulation, offering mechanical strength and biocompatibility when coupled with SA [27,28]. Poloxamers are of significant benefit for the stabilisation and solubilisation of products, whilst themselves having low toxicity [29].

Therefore, this study was designed to assess the dosage impacts of CDCA on the viability and function of pancreatic β-cells when incorporated into the manufactured microcapsules. This was conducted via the microencapsulation of MIN6 cells with various concentrations of CDCA. Also included in the formulations was SA, PLO and poloxamer. The resultant microcapsules were then assessed for their cell viability and distribution, inflammatory effects, glucose-induced properties and bioenergetic properties.

## 2. Materials and Methods

SA, PLO hydrochloride, poloxamer and CDCA were purchased from Sigma Chemical Co. (St. Louis, MO, USA). The MIN6 pancreatic β-cell line was kindly provided by Dr. Jun-ichi Miyazaki (Osaka University, Osaka, Japan). Excipients were utilised in formulations 1–4 to encapsulate MIN6 β-cells and formed within the gelation batch (2% CaCl_2_). CaCl_2_ dihydrate was purchased from Scharlab S.L (Sentrnenat, Spain). All four stock formulations consisted of SA (1.5%), PLO (2.5%), poloxamer (4%) and CDCA (0, 0.5, 3 and 8%).

### 2.1. Microcapsule Production

Microcapsules were produced using the BÜCHI-based microencapsulating system (BÜCHI Labortechnik, Flawil, Switzerland) connected to a flow-vibrational nozzle, with a built-in concentric unit [26,30,31,32]. The MIN6 pancreatic β-cells were processed through the internal nozzle whilst the stock mixtures of formulations were processed through the outer nozzle; even distribution was ensured by the concentric nozzle system. Microcapsules were captured in a gelation bath, consisting of 2% CaCl_2_ which was prepared by adding the appropriate weight of powder to ultrapure water. Formulations 1 through 4 were produced (F1 to F4), with the difference between the four being the CDCA concentration. F1 had no CDCA, F2 0.5% CDCA, F3 3% CDCA and F4 8% CDCA.

### 2.2. Microscopy

Both cell and CDCA distribution within the microcapsules were assessed via staining and subsequent confocal microscopy. Microcapsules were stained with CellTrace carboxyfluorescein succinimidyl ester (CFSE) from a Cell Proliferation Kit (Life Technologies, Carlsbad, CA, USA). CFSE was used to stain the encapsulated MIN6 cells. Microcapsule CDCA distribution was assessed via the conjugation of CDCA with the fluorescent compound tetramethylrhodamine isothiocyanate (TRITC). In a controlled environment, the stained cells and CDCA were viewed and assessed with an UltraVIEW Vox spinning disc microscope (Perkin Elmer, Waltham, MA, USA) which was fitted with a Yokogawa CSU-X1 confocal scanning unit (Perkin Elmer Waltham, MA, USA) as per our laboratory’s established methods [33,34].

### 2.3. Microencapsulated Cell Viability

Cell viability was assessed using a Countess Cell Counter chamber (Invitrogen, Seoul, South Korea), and in a Countess Automated Cell Counter (Invitrogen, Seoul, South Korea) which can assess cell viability and cell counts. The control used during these assessments was unencapsulated MIN6 cells. A detailed protocol of the method used can be seen in other published works [35,36]. Simply, post-encapsulation, the microcapsules undergoing this analysis were ruptured, then stained with trypan blue. In the process, the dilution factor, cell count of encapsulated cells, and viable cells post-rupture of microcapsules were accounted for to determine cell viability.

### 2.4. Glucose-Induced Assessments of Microencapsulated Cells

Several assays were conducted to assist in the assessment of the microencapsulated cells’ biological activity induced by glucose. Assays including glucose-induced cellular insulin release studies with an ultrasensitive mouse insulin ELISA kit (Mercodia Cooperation, Uppsala, Sweden) and MTT studies were used to assess glucose-induced viability. MTT reagent, 3-(4,5-dimethylthiazol-2-yl)-2,5-diphenyltetrazolium bromide (Sigma Chemical Co. St. Louis, MO, USA) was utilised in a validated method, previously established by the laboratory, allowing accurate assessment of cell viability in microcapsules without having to rupture the capsules [33,34]. Measurements of antioxidant activity were taken using a plate reader (Enspire, PerkinElmer, Waltham, MA, USA) for fluorescence, with increased fluorescent readings indicating increased oxidative stress, and therefore, decreased antioxidant activity. Oxidative stress measurements were taken from oxidized radical species following incubation with a mixture of dichloro-dihydro-fluorescein diacetate and 2,2′-azobis-2-methyl-propanimidamide dihydrochloride [25,37,38]. Assessments were taken under glycaemic and hyperglycaemic conditions.

### 2.5. Cytokine Assessments

Pro and anti-inflammatory cytokine assessments were conducted with Cytokine Bead Array (CBA) technologies (BD Biosciences, San Jose, CA, USA). Cytokine biomarkers tumor necrosis factor (TNF)-α, interferon (IFN)-γ, interleukin (IL)-6, IL-1β and IL-10 were examined. Briefly, the methodology involved aliquots of microcapsules containing cells being prepared according to the manufacturer’s protocols for use with the BD Flex Sets. The analysis was conducted using an Attune Acoustic Flow Cytometer (Life Technologies, Carlsbad, CA, USA) [39].

### 2.6. Bioenergetic Assessments

Mitochondrial activity and bioenergetic assessments were carried out with the Seahorse Flux Analyzer XF 96 (Seahorse Biosciences, Santa Clara, CA, USA). Assessments included ATP production, respiratory and glucose-induced assessments, measured via a fluorescent biosensor. All aforementioned analysis were conducted via our established methods, with controls being unencapsulated MIN6 pancreatic β-cells [33,39].

### 2.7. Statistical Analysis

Statistical analysis was conducted with Prism^®^ version 8.0 software (GraphPad Software, Inc., La Jolla, CA, USA), using a one-way ANOVA analysis of variance. Experiments were conducted in triplicate (*n* = 3). Statistical significance was set at *p* < 0.05. Data is presented as Mean ± SEM.

## 3. Results

### 3.1. Cell and CDCA Distribution

**Figure 1:**
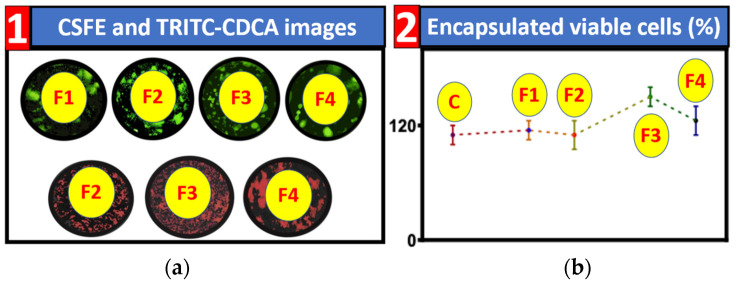
CFSE and TRITC-CDCA microscopic images and Encapsulated cell viability. (**a**). Top row Formulations 1 to 4 CFSE stained indicating distribution of MIN6 pancreatic β-cells in green. Bottom row TRITC conjugated CDCA images showing CDCA distribution in red. (**b**). Graph of % of viable cells post-encapsulation. The control is unencapsulated MIN6 pancreatic β-cells C. Graphed as as Mean ± SEM.

Figure 1a shows the distribution of cells and CDCA in the microcapsules via CFSE and TRITC. The top row of images shows the MIN6 pancreatic β-cells stained green, and their location throughout the capsules. F2 and F4 demonstrate the most cells with more even distribution throughout the microcapsule. F1 and F4 appear to show more clumping, and cells are distributed over a smaller area, although they do appear to be in all layers of the microcapsules’ matrix. In total, all microcapsules do contain MIN6 cells, distributed throughout the microcapsule matrix. In terms of CDCA, this is indicated by red staining due to TRITC conjugation with CDCA in the second row of images. F1 is not present as it does not contain any CDCA. The images indicate that CDCA is well distributed throughout the matrix of the microcapsules, showing a strong red colour throughout all three of the formulations which were tested. This is consistent with previous studies conducted using CDCA, in which results showed a similar distribution of CDCA throughout microcapsules [22]. As the aim is for evenly distributed microcapsules, all formulations show promise moving forward.

### 3.2. Cell Viability

Cell viability is indicated in Figure 1b. This is a measurement of the viability of MIN6 pancreatic β-cells within the microcapsules, with unencapsulated cells serving as the control. Results showed F3 to have the highest cell viability, when compared to the control and other formulations. The cell viability results demonstrate that microencapsulation does not decrease the viability of the cells, instead, particularly for F3, enhancing their environment and subsequently their viability. F1, without CDCA demonstrated a slight improvement of viability compared to the control, further reinforcing that the encapsulation process does not harm cells viability. Furthermore, F3, containing 3% CDCA offered the highest viability, followed by F4 which had 8% CDCA, demonstrating how idealistic concentrations of CDCA can improve cell viability of MIN6 pancreatic β-cells in comparison to microcapsules without CDCA and unencapsulated cells.

### 3.3. Glucose-Induced Viability and Oxidative Stress

**Figure 2:**
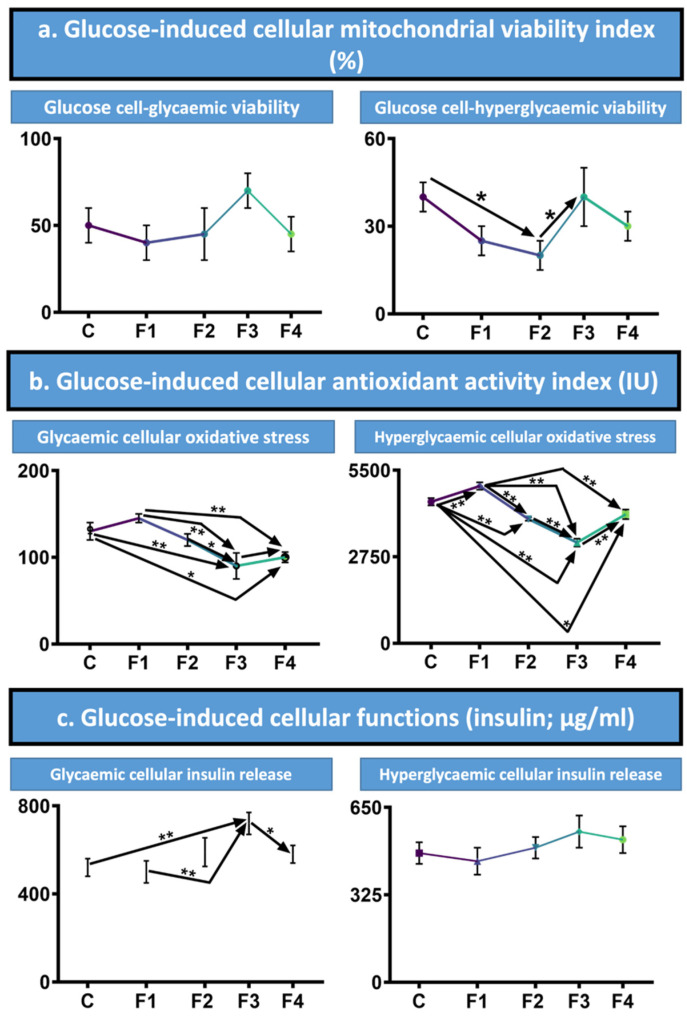
Glucose-induced viability, antioxidant activity and insulin release. (**a**). Glycaemic and hyperglycaemic conditions cell viability (%). (**b**). Glycaemic and hyperglycaemic antioxidant activity as oxidative stress (IU). (**c**). Glycaemic and hyperglycaemic cellular insulin release (µg/mL). Assessed on formulations 1 to 4 with unencapsulated MIN6 pancreatic β-cells as the control (C). Presented as Mean ± SEM, with arrows used to denote any statistical significance between data points. * *p* < 0.05, ** *p* < 0.01.

The glucose-induced cellular mitochondrial viability, as measured via MTT assays, is summarised in Figure 2a. MIN6 pancreatic β-cell viability was assessed under glycaemic conditions to assess how these conditions affect the encapsulated cells, with non-encapsulated cells serving as the control. The control and test formulations of microcapsules were treated with 25 mmol/L glucose for 24 h. Under these conditions, the viability of the negative control was 50% ± SEM, with F1 viability was 40% ± SEM and F2 and F4 were 45% ± SEM, indicating a decrease in viability in comparison to the control. However, F3 performed better under such conditions, with a viability of 70% ± SEM, indicating these microcapsules have the greatest ability, of those assessed, to withstand such conditions and protect the cells. To add to these results, hyperglycaemic conditions of 35 mmol/L glucose were also conducted. Once again, F3 performed best, equalling the results of the negative control. F2 survival was decreased statistically significantly (*p* < 0.05) compared to the control and F3, with viability of 20% ± SEM in comparison to the control and F3′s 40% ± SEM. F1 and F4’s viability from treatment with 35 mmol/L of glucose fell between F2 and F3.

Figure 2b also highlights the cellular antioxidant index, determined under the same conditions as above. In glycaemic conditions, with treatment of 25 mmol/L of glucose, the oxidative stress was recorded. In comparison to the control, F1 had a higher oxidative stress, whilst all other formulations decreased oxidative stress. Of the CDCA formulations, F3 had the lowest oxidative stress, followed by F4 then F2. F3 and F4 had a statistically significantly decrease in oxidative stress compared to the control (*p* < 0.01) and (*p* < 0.05) respectively; whilst F3 and F4 both had lower in oxidative stress than F1 (*p* < 0.01). The oxidative stress results from F3 were also below that of F2 (*p* < 0.05). F3 demonstrated the lowest oxidative stress, suggesting that this formulation is able to assist in alleviation of the oxidative stress induced via the glycaemic conditions, protecting the MIN6 pancreatic β-cells that were encapsulated. Further adding to this, all formulations containing CDCA had a decreased oxidative stress in comparison to the negative control of unencapsulated MIN6 pancreatic β-cells, indicating the protective effects of CDCA on the cells when encapsulated.

Under hyperglycaemic conditions, in which capsules were treated with 35 mmol/L of glucose, oxidative stress was assessed. Similar to the glycaemic conditions, F1 had an increased oxidative stress compared to the control (*p* < 0.01), with all other formulations having a lower oxidative stress. The overall levels of oxidative stress were also higher in these conditions, with the control being 4500 IU compared to 130 IU at 25 mmol/L glucose. From the CDCA formulations, F3 had the lowest oxidative stress, followed by F2 and then F4. F2 and F3 had statistically significantly lower oxidative stress than the control and F1 (*p* < 0.01), as was F4 to the control and F1, (*p* < 0.05) and (*p* < 0.01) respectively. In addition, F3 had oxidative stress results statistically lower than both F2 and F4, (*p* < 0.01). These results support those of the 25 mmol/L glucose studies, with idealistic concentrations of CDCA offering a protective barrier against oxidative stress from hyperglycaemic conditions. This is also consistent with work previously conducted by the laboratory [40].

### 3.4. Insulin Release

Insulin release was also measured under glycaemic conditions of 25 mmol/L, with unencapsulated MIN6 pancreatic β-cells serving as the control. F1 had a decreased insulin release, whilst F2, F3 and F4, all containing CDCA, increased insulin release, compared to the control. Overall, F3 released the most insulin, followed by F2 then F4. F3 had a statistically increased insulin release compared to F1 and the control, (*p* < 0.01); as well as to F4, (*p* < 0.05). Under hyperglycaemic conditions (treated with 35 mmol/L glucose), all formulations but F1 had an increased release of insulin compared to controls. F3 had the highest insulin release, followed by F4 then F2. Overall, these results demonstrate the benefit of CDCA in improving the insulin release in response to glucose by pancreatic MIN6 cells.

### 3.5. Glycaemic-Induced Cytokine Production

**Figure 3:**
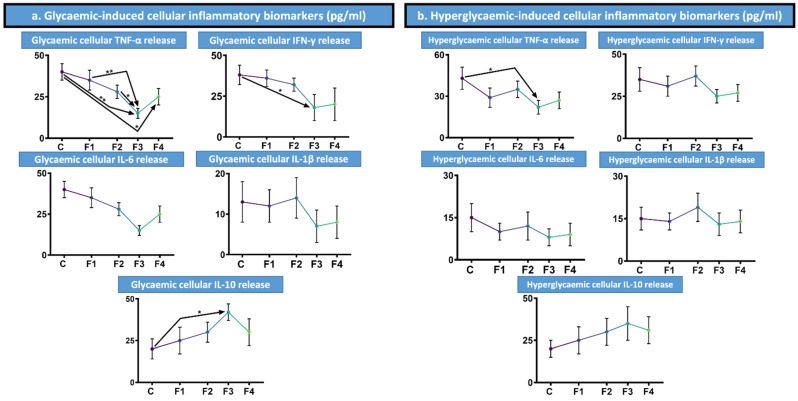
Cytokine biomarker detection in glycaemic and hyperglycaemic conditions. (**a**). Glycaemic-induced pro-inflammatory biomarker release TNF-α, IFN-γ, IL-6, and IL-1β; and anti-inflammatory IL-10 (pg/mL). (**b**). Hyperglycaemic-induced pro-inflammatory biomarker release TNF-α, IFN-γ, IL-6, and IL-1β; and anti-inflammatory IL-10 (pg/mL). Assessed on formulations 1 to 4 with the control being unencapsulated MIN6 pancreatic β-cells (C). Presented as Mean ± SEM, with arrows used to denote any statistical significance between data points. * *p* < 0.05, ** *p* < 0.01.

Figure 3 showed that under glycaemic conditions, of 25 mmol/L glucose, a range of pro-inflammatory biomarkers were measured, including TNF-α, IFN-γ, IL-6, and IL-1β; and the anti-inflammatory IL-10. Once again, the negative control was unencapsulated MIN6 pancreatic β-cells. In terms of TNF-α and IFN-γ, the CDCA formulations induced the lowest release, with the control having the highest. Statistically, F3 was lower than the control and F1, (*p* < 0.01); as well as F2 and F4, (*p* < 0.05) for TNF-α biomarker. For these two pro-inflammatory biomarkers, the CDCA formulations reduced their presence. This suggests that CDCA has anti-inflammatory effects against these cytokines for the MIN6 cells, reducing apoptosis and improving growth conditions. For IFN-γ, F3 had a statistically lower pro-inflammatory biomarker detection than the control, (*p* < 0.05). For biomarkers IL-6, and IL-1β, F3 was lowest, followed by F4, F1 then F2 and the control. These cytokine results further build on the theory that CDCA has anti-inflammatory effects based upon F3 and F4’s results. Whilst F2 had a higher pro-inflammatory effect, this was not statistically significant compared to the control. F2 only contained 0.5% CDCA compared to the 3% and 8% of F3 and F4 suggesting a dose dependent response. Anti-inflammatory biomarker IL-10 further supports the proposed anti-inflammatory effects of CDCA, with the highest cytokine detection from F3, followed by F4 and F2, F1 then the control. F3 was also statistically significantly higher than the control, (*p* < 0.05). These findings are consistent with our previous results [35,36].

### 3.6. Hyperglycaemic-Induced Cytokine Production

**Figure 4:**
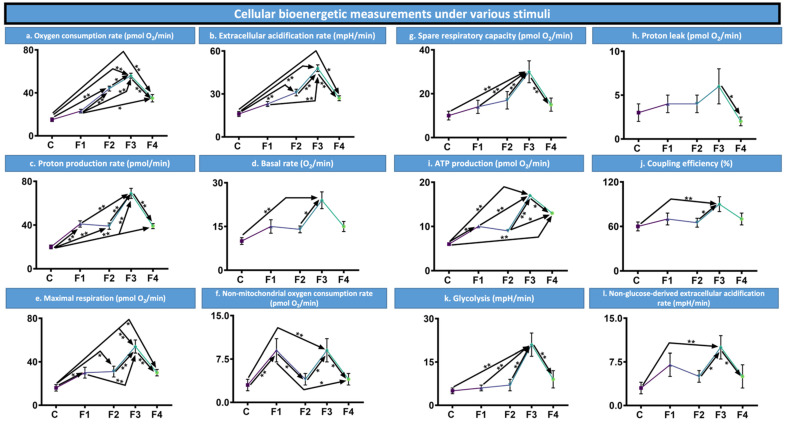
Cellular bioenergetic measurements under various stimuli. (**a**). Oxygen consumption rate (pmol O_2_/min). (**b**). Extracellular acidification rate (mpH/min). (**c**). Proton production rate (pmol/min). (**d**). Basal rate (O_2_/min). (**e**). Maximal respiration rate (pmol O_2_/min). (**f**). Non-mitochondrial oxygen consumption rate (pmol O_2_/min). (**g**). Spare respiratory capacity (pmol O_2_/min). (**h**). Proton leak (pmol O_2_/min). (**i**). ATP production rate (pmol O_2_/min). (**j**). Coupling efficiency (%). (**k**). Glycolysis (mpH/min). (**l**). Non-glucose-derived extracellular acidification rates (mpH/min). Assessed on formulations 1 to 4 with unencapsulated MIN6 pancreatic β-cells as the control (C). Presented as Mean ± SEM, with arrows used to denote any statistical significance between data points. * *p* < 0.05, ** *p* < 0.01.

A range of cytokines were measured under hyperglycaemic conditions of 35 mmol/L of glucose for pro-inflammatory TNF-α, IFN-γ, IL-6, and IL-1β; and anti-inflammatory IL-10, with the control being unencapsulated MIN6 pancreatic β-cells (Figure 4). F3 and F4 had the lowest production of all anti-inflammatory biomarkers tested, indicating the anti-inflammatory properties of CDCA, including at such hyperglycaemic conditions. Statistically, F3 had a lower production of pro-inflammatory TNF-α compared to the control, (*p* < 0.05). In terms of the anti-inflammatory IL-10, the highest rate was detected by F3, followed by F4, F2, F1 and finally the control with the lowest production. Such results demonstrate the anti-inflammatory ability of CDCA, which is beneficial to the encapsulated MIN6 pancreatic β-cells, assisting in supporting their growth and functionality with such anti-inflammatory conditions, including in hyperglycaemic conditions [35,36].

### 3.7. Bioenergetic Measurements

A range of bioenergetic measurements were taken under a variety of stimuli, including on the control samples (Figure 4). These measurements included assessments of oxygen consumption rates (pmol O_2_/min), basal oxygen rates (O_2_/min), maximal respiration rates (pmol O_2_/min), non-mitochondrial oxygen consumption rates (pmol O_2_/min), spare respiratory capacity (pmol O_2_/min) and ATP production rates (pmol O_2_/min). In addition to extracellular acidification rates (mpH/min), proton production rates (pmol/min), rates of proton leaks (pmol O2/min), coupling efficiency (%), rates of glycolysis (mpH/min) and non-glucose-derived extracellular acidification rates (mpH/min).

Overall, F3 performed best from all formulations and the control under all stimuli. In all measurements, aside from the proton leak data set, the control performed worst, offering the lowest results. This indicates that microencapsulation positively impacts the MIN6 pancreatic β-cells and their bioenergetic measurements. Furthermore, as mentioned, F3, containing 3% CDCA, consistently outperformed the other formulations and control; indicating that CDCA assists in cellular functions and subsequent bioenergetic measurements, when used at the correct concentration, as demonstrated by these concentration-dependent results.

## 4. Discussion

### 4.1. Cell and CDCA Distribution and Cell Viability

Results of cell distribution demonstrated that MIN6 cells were distributed within the matrix of the microcapsules for all four formulations, with these results indicating the successful microencapsulation of the cells. Cell distribution in the microcapsule is a paramount element, with cells idealistically distributed throughout the microcapsule, but not within the outer layers in order to offer them protection from the host’s environment, shielding from immune responses [7,41]. CDCA was also found to be distributed throughout the microcapsules, with this distribution likely to allow the BA CDCA to interact with the encapsulated cells, permitting an impact on cell viability [33].

The overall increased survival of cells encapsulated with higher concentrations of CDCA was akin to results from other studies, in which CDCA addition optimised cell survival compared to controls without CDCA [42]. However, the research presented here also improved on previous works, demonstrating an overall higher cell viability percentage, indicating the improvements of the study processes, including encapsulation. The formulation-dependent changes to viability also indicate the impacts of CDCA at various concentrations, and the necessity for the development of BA-based microcapsules to have the appropriate concentrations of BAs within.

### 4.2. Glucose-Induced Viability and Oxidative Stress

Cell viability under glycaemic and hyperglycaemic conditions was shown to be formulation dependent, with changes in results due to the presence and concentration of CDCA within. The potential for BAs to improve cell viability has previously been described in studies by this research group, with the improvements also varying between formulations. As discussed above from cell viability studies in the absence of glucose stimuli, the variations in viability under glucose stimuli are also likely to be due to the interactions between CDCA and the encapsulated β-cells [25,40,43].

Both glycaemic and hyperglycaemic assessments of oxidative stress demonstrated the antioxidant effects of CDCA, with these benefits still being present when CDCA is encapsulated. Overall, results showed a reduction in the oxidative stress measurements from the cells, indicating CDCA’s antioxidant properties. BAs, including CDCA, are known activators of FXR, with FXR regulating homeostasis and also showing cytoprotective effects [44]. The activation of FXR has previously been shown to reduce oxidative stress. Furthermore, Gai et al. induced hypoxia in proximal tubular cells, demonstrating that cells treated with 6-ethyl-CDCA, which is CDCA with an alkyl substitution, had reduced levels of hypoxia-induced oxidative stress. Their research demonstrated this reduction to be from 6-ethyl-CDCA activating FXR, resulting in the induction of nuclear factor erythroid 2–related factor 2 (Nrf2), causing the activation of Nrf2-antioxidative pathways [45]. Nrf2 is a transcription factor which has been shown to offer oxidative resistance via the regulation of oxidant levels and signalling. When activated, Nrf2 plays roles inducing the expression of signalling proteins and enzymes, resulting in its impact on drug metabolism, signalling of oxidants, and antioxidant defensive factors [46]. Previously, Nrf2 antioxidant responses have been demonstrated to be activated by BAs, including CDCA, inducing Nrf2 target genes; hence, CDCA’s role in reducing oxidative stress [47].

### 4.3. Glucose-Induced Insulin Release

Further adding to the described interactions between CDCA and the MIN6 cells, the interaction is likely to have influenced the insulin release from the cells. This is consistent with the results from this study, with the overall addition of CDCA improving insulin release. Such improvement in insulin release is similar to previous studies, including one in which CDCA was found to increase insulin production, with CDCA having a biological effect on β-cells [42]. In addition, results from a study on the oral ingestion of CDCA demonstrated the BA to improve insulin sensitivity, further enhancing the benefits of the inclusion of CDCA in the microcapsules [48].

Previously published work by Shihabudeen et al. demonstrated that in palmitate- treated cells stimulating insulin resistance, treating the cells with CDCA resulted in improved insulin sensitivity. Furthermore, studies with rats who were fed high fat diets and developed a resistance to insulin, showed treatment with CDCA to reverse their impaired insulin tolerance [49]. Previous work has highlighted that deficiencies in FXR cause an increase in resistance to insulin in both muscle and the liver [50]. Hence, an agonist of FXR, such as CDCA, may be beneficial to improve insulin sensitivity and potentially be utilised in the treatment of diabetes mellitus as a therapeutic agent. Cipriani et al. investigated the effects of the treatment with 6-ethyl-CDCA, on fa/fa rats, with CDCA activating FXR. The authors concluded that the activation of FXR via CDCA resulted in a reversal of the insulin resistance, resulting in normalized plasma insulin levels and insulin sensitivity [51].

### 4.4. Cytokine Biomarkers

Cytokine results showed all formulations with CDCA to increase the measurements of the anti-inflammatory IL-10, whilst reductions in pro-inflammatory biomarkers were most present in F3 and F4, with high concentrations of CDCA, indicating a dose-dependent effect. Overall, the formulations demonstrated CDCA to have an anti-inflammatory effect. Previous studies researching the effects of CDCA have shown the BA to exhibit anti-inflammatory effects. Such effects are the result of the activation of FXR, which demonstrates anti-inflammatory activities, suppressing pro-inflammatory genes, whilst simultaneously enhancing those which are anti-inflammatory and reducing overall inflammation [21,49]. In order to have successful microcapsules in vivo, they must be biocompatible. One key factor for this is ensuring the biomaterials utilised in the microcapsules won’t induce an inflammatory reaction. Also required is the reduction of pro-inflammatory molecules which may be released by the encapsulated cells, potentially resulting in microcapsule overgrowth with immune cells and fibroblasts, on the surface or in the vicinity of the microcapsules [41,52]. Hence, the reductions in pro-inflammatory cytokines and increase in the anti-inflammatory marker from F3 and F4 show promise for reduced inflammatory responses.

### 4.5. Bioenergetic Measurements

Bioenergetic measurements were shown to be CDCA concentration-dependent, with results varying between formulations. Overall, F3 increased all bioenergetic measurements to the control. Previous studies have demonstrated that the increases and improvements of bioenergetic measurements, particularly glycolysis, maximal respiration, basal respiration and ATP production are likely to be a dose-dependent response to the addition of CDCA resulting in intracellular activation, as well as the enhanced inner-membrane mitochondrial manufacture of ATP [42,53]. The results suggest that CDCA positively impacts the biological activity of the β-cells. Increases in ATP production rates are also likely to have positively influenced the insulin production results, with pancreatic β-cells’ metabolic changes influencing the ATP-sensitive potassium channel which leads to the secretion of insulin [54,55].

## 5. Conclusions

This study has indicated the numerous benefits of CDCA in cell encapsulation, confirming that the known positive impacts of CDCA remain present when encapsulated. Results demonstrated how CDCA improved cell viability, including under glycaemic and hyperglycaemic conditions. Insulin release was also improved, and CDCA demonstrated anti-inflammatory effects. This study, combined with prior research of CDCA demonstrate the wide nature of benefits associated with its inclusion in manufactured capsules, including the potential of further benefits in vivo. Overall, F3 containing 3% CDCA performed best in this study, indicating a concentration dependent effect of CDCA in encapsulation and the requirement for the inclusion of idealistic concentrations in future research.

## Data Availability

The data presented in this study are available on request from the corresponding author. The data are not publicly available due to author property agreements.
